# The Effect of the Fresh Latex Ratio on the Processing and Dynamic Properties of Bio-Coagulated Natural Rubber

**DOI:** 10.3390/polym17111435

**Published:** 2025-05-22

**Authors:** Jianwei Li, Yun Li, Li Ding, Honghai Huang, Tuo Dai, Liguang Zhao, Yingguang Xu, Fan Wu, Hongxing Gui

**Affiliations:** 1 Hainan Natural Rubber Technology Innovation Center, Rubber Research Institute, Chinese Academy of Tropical Agricultural Science, Haikou 571101, China; 18272733025@163.com (J.L.); gdzjding@163.com (L.D.); honghaihuang2009@163.com (H.H.); catasdaituo@163.com (T.D.); zhaoliguang@catas.cm (L.Z.); crucxjsdjd@163.com (F.W.); 2 Mengla Tianye Rubber Sales Co., Ltd., Xishuangbanna 666100, China; 15125550466@163.com; 3 Hainan Academy of Inspection and Testing, Haikou 571101, China; 18976653393@163.com

**Keywords:** variety, fresh latex, ratio, processing property, physical and mechanical property

## Abstract

Natural rubber is a widely used biological polymer material because of its excellent comprehensive performance. Nevertheless, the performance of domestic natural rubber cannot meet the requirements for high-end products such as aviation tires, which has become a constraint on the innovation and upgrading of high-end manufacturing enterprises and the enhancement of global competitiveness in China. To solve the bottleneck problem of natural rubber processing technology, this study systematically analyzed the effects of different varieties of fresh latex ratios on the processing and dynamic properties of bio-coagulated natural rubber. By mixing PR107 and Reyan72059 fresh latex with Reyan73397 fresh latex according to proportion, the fresh latex was coagulated by enzyme-assisted microbials, and the effects of the fresh latex ratio on physical and chemical indexes, molecular weight distribution, vulcanization characteristics, processing properties, cross-link density and physical and mechanical properties of the natural rubber were analyzed. The results showed that the aging resistance of natural rubber coagulated with enzyme-assisted microbial decreased, and the aging resistance of natural rubber increased with the increase in the mixing ratio of PR107 and Reyan72059 fresh latex. The proportion of high molecular weight of the natural rubber coagulated with the enzyme-assisted microbial increased, and the fresh latex mixing had little effect on the molecular weight distribution curve. Under the carbon black formulation, the CRI of the enzyme-assisted microbial coagulated natural rubber compound was relatively larger. Under the same strain conditions, the H-3 compound (PR107:Reyan72059:Reyan73397 = 1:1:3) had the best viscoelasticity and the least internal resistance of rubber molecules. In addition, the cross-link density, tensile strength, elongation at break, and tear strength of H-3 vulcanized rubber were the largest, improved by 23.08%, 5.32%, 12.45% and 3.70% compared with the same H-2 vulcanized rubber. In addition, the heat generation performance was reduced by 11.86%, and the wear resistance improved.

## 1. Introduction

Natural rubber is a biological polymer material prepared from fresh latex through the processes of coagulation, pressure creping, washing, and drying [1,2], and it is irreplaceable in the field of high-end products such as aviation tires, high-speed railway vibration damping pads, and heavy-duty truck tire treads due to its excellent comprehensive performance. However, fresh rubber latex is affected by a combination of factors such as soil, climate, season, and variety during the synthesis of rubber tree milk tubes, which makes the quality of natural rubber vary significantly. Among these, the rubber tree variety is one of the key factors determining the quality of natural rubber. Studies have shown that there are great differences between different rubber tree varieties in terms of rubber hydrocarbon content, rubber particle size, non-rubber components, molecular weight size and distribution, gel structure, and other aspects [3,4,5,6,7,8,9,10,11,12,13]. At present, China’s existing high-performance natural rubber processing technology cannot meet the rubber requirements of high-end products, resulting in long-term dependence on imports of high-end products, which seriously affects the security of the supply of national strategic materials and industrial raw materials.

At present, high-performance natural rubber in China generally adopts a microbial coagulation of fresh latex, which avoids the addition of ammonia and acid to the fresh latex, and reduces environmental pollution, equipment corrosion, and human health damage and other issues. Moreover, the performance of the natural rubber prepared is superior to that of the natural rubber solidified with formic acid, but it still cannot match the imported grade I smoked sheet rubber [14]. Subsequently, the use of enzyme-assisted microorganisms to coagulate fresh latex has significantly increased the physical and mechanical properties of the prepared natural rubber [15], further enhancing the comprehensive performance of domestic natural rubber. However, the comprehensive performance still failed to meet the requirements of special rubber for high-end products. To solve the bottleneck problem of processing technology for domestic high-performance natural rubber, the effects of fresh latex ratio on the processing and dynamic properties of bio-coagulated natural rubber were studied. PR107 and Reyan72059 fresh latex were added to Reyan73397 fresh latex according to the ratio, and enzyme-assisted microbial coagulation was used to coagulate the mixed fresh latex. The effects of fresh latex ratio on the physical and chemical indexes, molecular weight size and distribution of raw rubber, vulcanization characteristics and processing properties of compound, physical and mechanical properties, heat generation performance and wear resistance of vulcanized rubber were analyzed. The corresponding relationship between fresh latex ratio and natural rubber performance was explored, and the optimal ratio of different varieties of fresh latex was screened. Based on the in-depth study of the effect of fresh latex ratio on the processing and dynamic performance of natural rubber, the comprehensive performance of natural rubber can be significantly improved by optimizing the ratio of fresh latex, so that its product performance can meet the demand for rubber used in aviation tires, high-speed railway vibration damping pads and other high-end products.

## 2. Materials and Methods

### 2.1. Materials and Instruments

PR107 and Reyan72059 fresh latex (dry rubber content 36.5%), Reyan73397 fresh latex (dry rubber content 35.0%), and microbial strains were provided by the Rubber Research Institute of the Chinese Academy of Tropical Agricultural Sciences, Haikou, Hainan, China; alkaline protease (200,000 µ/g) was provided by Beijing Coolaibo Science and Technology Co., Ltd., Beijing, China; and formic acid, zinc oxide, stearic acid, sulfur, carbon black, etc., are commercially available industrial compounds.

The equipment was sourced as follows: Electronic balance, Orebo FA2204 B. Rotorless rheometer, Goodtechwill testing machines (Qingdao) Co., Ltd. (Qingdao, China) M-3000A. Open-type rubber mixing machine, Guangdong Zhanjiang Machinery Factory JTC-752. Platen vulcanizing press, Guangdong Chaozhou Hongqiao Rubber Machinery Factory (Zhanjiang, China) 250KWXLB-D. Tensile testing machine, Goodtechwill testing machines (Qingdao) Co., Ltd. (Qingdao, China) AI-7000-SGD1. Nuclear magnetic resonance (NMR) cross-link density analyzer, Germany IIC company (Engen, Germany) XLDS-15. Rubber process analyser (RPA), Montech (Buchen, Germany) D-RPA3000. Electromagnetic dynamic mechanical testing system, Kyle Measurement and Control Technology Co., Ltd. (Wuhan, China) M-3000. Akron abrasion testing machine, Goodtechwill testing machines (Taiwan) Co., Ltd., GT-7012-A.

### 2.2. Methods

#### 2.2.1. Natural Rubber Preparation

The dry rubber content of fresh latex was diluted to 23%, and the fresh latex was mixed according to the ratios in Table 1. The mixed fresh latex was stirred, and the coagulant was added uniformly. After the fresh latex had completely coagulated, the coagulum underwent maturation for 2 days, followed by creping, washing, and sheet hanging for 3 days, and then drying to prepare natural rubber samples. The samples were numbered H-1, H-2, H-3, H-4, and H-5. Acid coagulation and enzyme-assisted microbial coagulation of a single variety of natural rubber were used as control samples.

#### 2.2.2. Preparation of Natural Rubber Compound

The natural rubber compound was prepared by taking 500 g raw rubber and using a carbon black formula (Table 2) according to the NY/T 1403-2007 [16] standard.

**Figure 1:**
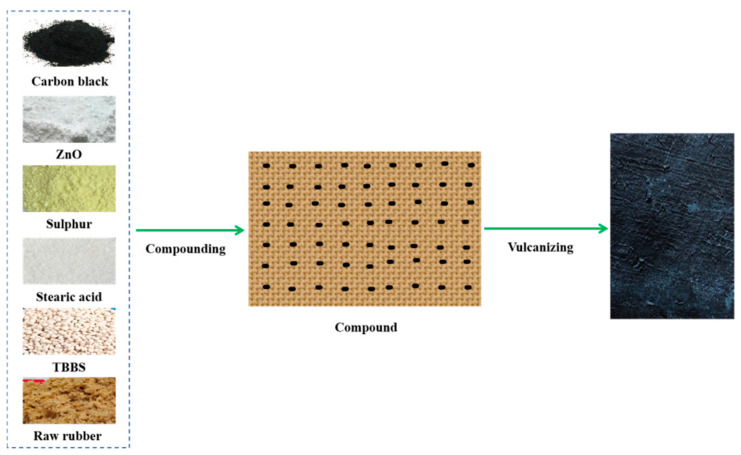
The preparation process of natural rubber vulcanized rubber.

The compounds were vulcanized by using a platen vulcanizing press at 140 °C for 30 min to prepare vulcanized rubber test pieces (Figure 1). Heat generation and wear samples were prepared at 140 °C for t_90_ + 10 min.

### 2.3. Testing and Analysis

(1) The plasticity initial value (P_0_), plastic retention rate (PRI), and Mooney viscosity of natural rubber were determined according to GB/T 3510-2006 [17], GB/T 3517-2014 [18], and GB/T 1232.1-2016 [19] standards.
(2) The molecular weight distribution of natural rubber was determined by gel permeation chromatography. The test conditions were as follows: the sample concentration was 2 mg/mL, the mobile phase was tetrahydrofuran, and the standard sample was polystyrene.
(3) The vulcanization characteristics of the natural rubber compound were determined by rotorless rheometer, with the following test conditions: test temperature of 160 °C, test time of 20 min, oscillation frequency of 1.7 Hz, and amplitude of ±0.5°.
(4) The processing properties of the natural rubber vulcanized rubber were determined by rubber processing analysis. Strain scanning: frequency 0.5 Hz, temperature 100 °C, strain range 0~100%.
(5) The cross-link density of the natural rubber vulcanized rubber was determined by a nuclear magnetic resonance cross-link density analyzer. The test conditions were as follows: resonance frequency 22.00 MHz, magnet strength 0.35 Tesla, experimental temperature 35.00 ± 0.01 °C.
(6) The physical and mechanical properties of the natural rubber vulcanized rubber were determined by a tensile testing machine according to GB/T 528-2009 [20] and GB/T 528-2008 [21] standards, with a running speed of 500 mm/min.
(7) The heat generation performance and wear resistance of the natural rubber vulcanized rubber were determined according to GB/T 1687.3-2016 [22] and GB/T 1689-2014 [23] standards.

## 3. Results and Analysis

### 3.1. Physical and Chemical Indexes of Natural Rubber

P_0_ reflects the size of rubber plasticity, PRI reflects the strength of rubber aging resistance, and M_L(1+4)_ reflects the difficulty of rubber processing; the variation in physical and chemical indexes of natural rubber is shown below (Table 3). As can be seen from the table, the P_0_ and ML_(1+4)_ of the H-1 sample are the smallest, while PRI is the largest, indicating that the plasticity of acid-solidified natural rubber is large, and the processing performance and aging resistance are better. The changes in P_0_ and ML_(1+4)_ of the H-2~H-5 samples are small, while the PRI is gradually increasing, which indicates that the increase in the mixing ratio of PR107 and Hot Research 72059 fresh latex does not significantly affect the plasticity of natural rubber and the processing performance. The processing performance is not obvious, but it can significantly improve the aging resistance of natural rubber.

### 3.2. Molecular Weight Distribution of Natural Rubber

**Figure 2:**
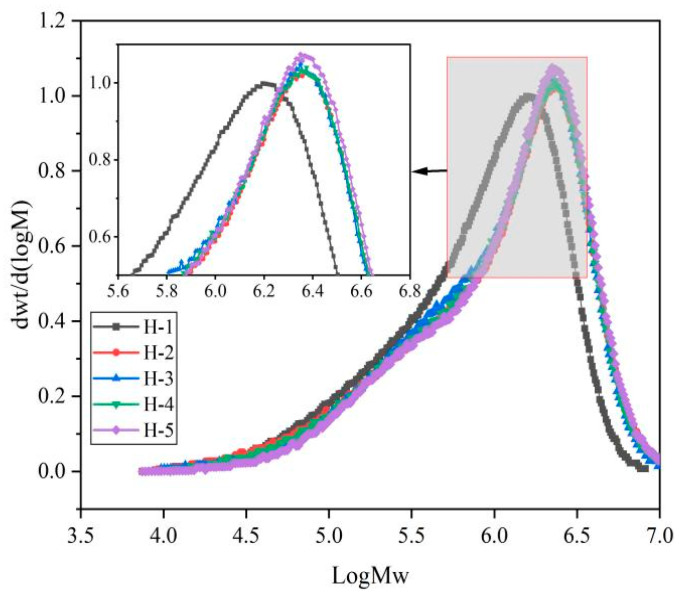
Molecular weight distribution of natural rubber.

The molecular weight distribution curve of natural rubber is shown below (Figure 2). As can be seen from the figure, the molecular weight distribution curves of all samples are roughly similar, showing a typical single-peak distribution, with a peak value in the range of 6.0~6.5, indicating that the number of samples in this region of molecular weight accounts for the largest proportion of the total number of samples, which means that the number of samples in this region of molecular weight is the largest. Compared with the H-1 sample, the molecular weight distribution curves of H-2~H-5 samples were shifted to the right; that is, the proportion of the high molecular weight part increased, indicating that the coagulation process had a significant impact on the molecular weight distribution curve of natural rubber, which was due to the cross-link of natural rubber networks being increased by enzyme-assisted microbial coagulation [24,25]. The peak values of H-2~H-5 samples were almost similar, indicating that the mixing of different varieties of fresh latex had little effect on the molecular weight distribution curve of natural rubber, which shows that the effect of the coagulation process on the molecular weight distribution of the natural rubber is greater than that when mixing fresh latex.

### 3.3. Vulcanization Characteristics of the Natural Rubber Compound

The change in vulcanization characteristics of natural rubber compounds is shown below (Table 4). In the table, the minimum torque (M_L_) reflects the fluidity of the compound at a specific temperature, the maximum torque (M_H_) reflects the modulus of the compound, and ∆M reflects the degree of cross-link of the rubber. The scorching time (t_10_) reflects the early processing safety of the rubber, the positive vulcanization time (t_90_) reflects the time required for the rubber to reach the optimum degree of vulcanization, and the CRI reflects the rate of vulcanization of the rubber [26,27,28]. As can be seen from the table, compared with the H-1 compound, the M_L_, t_10_, t_90_, and CRI of the H-2 compound are relatively larger, indicating that the fluidity, processing safety, optimum vulcanization time, and vulcanization rate of the acid-coagulated natural rubber compound are higher, which is due to the significant influence of the coagulation manner of the fresh latex on the vulcanization characteristics of the natural rubber compound [26]. With the increase in the mixing ratio of PR107 and Reyan72059 fresh latex, the ∆M and CRI of H-2~H-5 compounds first increased and then decreased, indicating that the cross-link degree and vulcanization rate of the rubber compound could be improved by mixing ratio of different varieties of fresh latex.

### 3.4. Processing Properties of the Natural Rubber Compound

**Figure 3:**
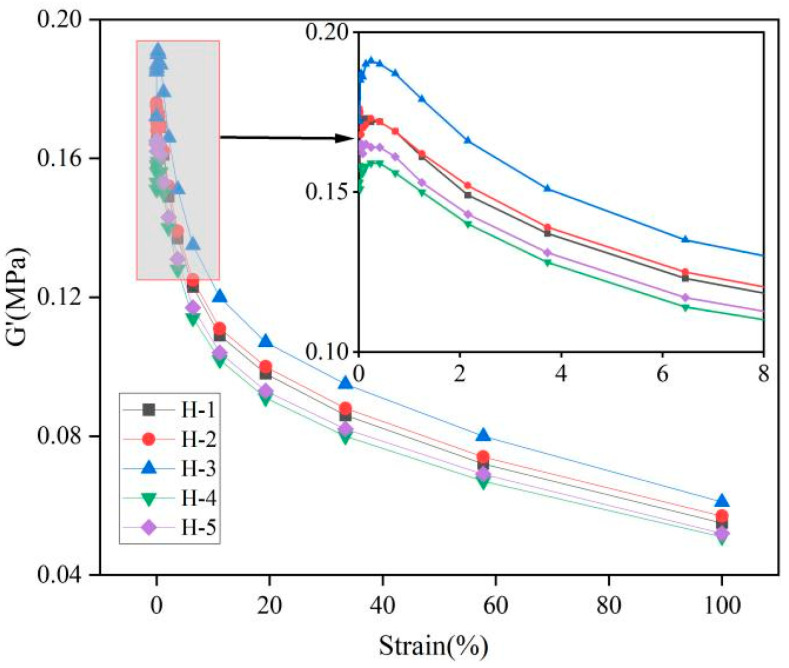
Responses of G′ against strain of natural rubber compound.

**Figure 4:**
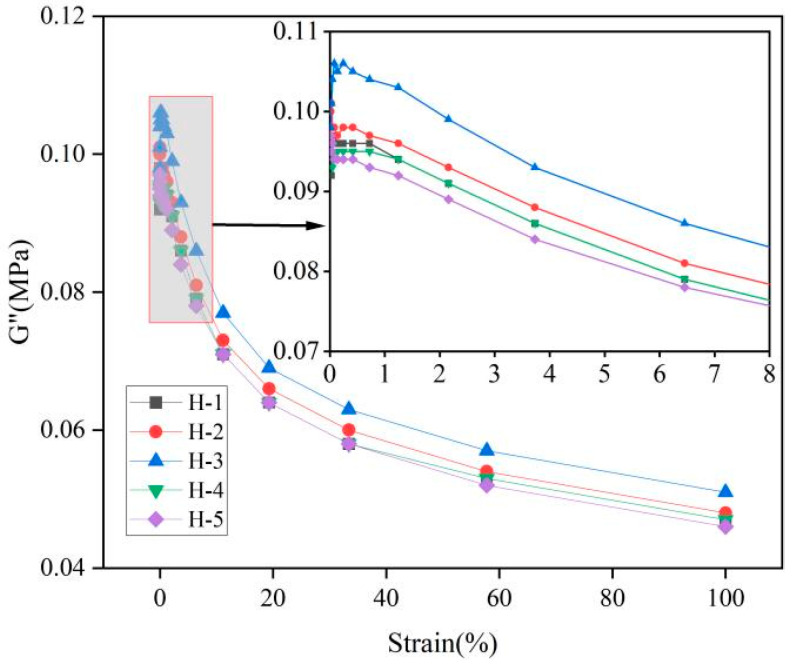
Responses of G″ against strain of natural rubber compound.

The changes in energy storage modulus (G′) and loss modulus (G″) of the natural rubber compound are shown as follows (Figure 3 and Figure 4). As can be seen from the Figures, at low strain, the G′ and G″ of the natural rubber compound are larger, indicating that the natural rubber compound has higher stiffness, elasticity, and energy dissipation capacity under low strain, because the carbon black filler network is not damaged under low strain, and only the orientation of the chain segments and the slip of the molecular chains occur [29]. With the gradual increase in strain, the G′ and G″ of the compound gradually decrease, and the rate of modulus change with strain also decreases, indicating that the elasticity of natural rubber blends decreases and the energy dissipation ability becomes poor, and the ability of the modulus to respond to strain is weakened, which is due to the carbon black filler network being destroyed under high strain, and the linear viscoelasticity gradually disappears [30]. Under the same strain condition, when the ratio of PR107, Reyan72059, and Reyan73397 fresh latex is 1:1:3, the G′ and G″ of the compound were the highest. With the increase in the addition amount of fresh latex of PR107 and Reyan72059, the G′ and G″ of the compound decrease, indicating that the appropriate mixing ratio of different varieties of fresh latex can improve the viscoelasticity of its compound rubber. This may be due to the mixing of different varieties of fresh latex changing the ratio of large and small rubber particles and the content of non-rubber components in natural rubber, etc., which increases the dispersibility of carbon black in the rubber, resulting in an increased interaction force between the carbon black and rubber [3,31,32,33].

**Figure 5:**
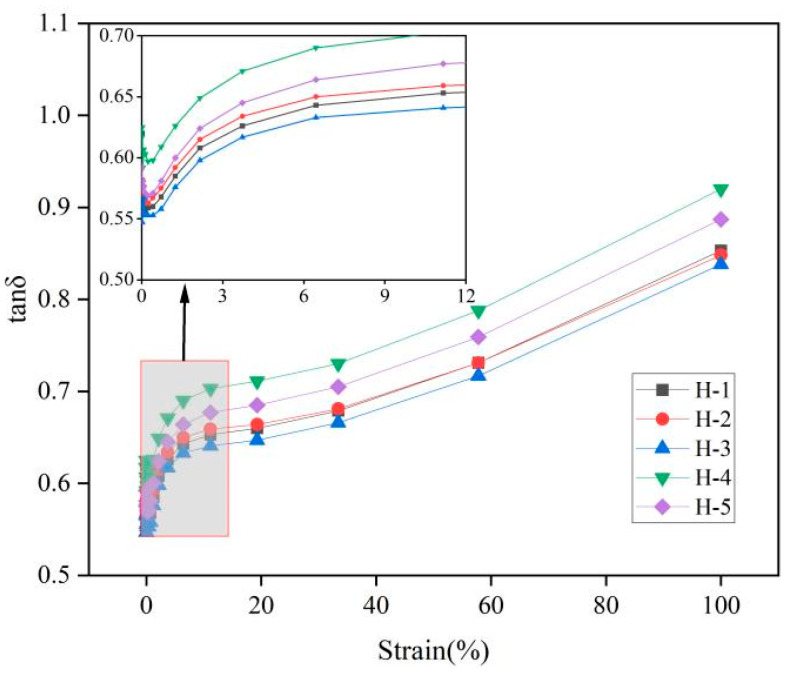
Responses of tanδ against strain of natural rubber compound.

The change in the natural rubber compound loss factor (tanδ) is shown below (Figure 5). Tanδ is the ratio of G″ to G′, which reflects the ratio of viscous to elastic behavior of the compound and the internal resistance of rubber molecules in viscous flow [30]. The higher tanδ indicates that with more significant viscous behavior and greater internal resistance of rubber molecules in viscous flow, more energy needs to be consumed to overcome this obstruction. The lower tanδ indicates that with more significant elastic behavior and less internal resistance of rubber molecules in viscous flow, less energy needs to be consumed to overcome this obstruction. As can be seen from the figure, tanδ increases with the increase in strain, and the ratio of the viscous behavior to the elastic behavior of natural rubber compound gradually increases, indicating that with the growth of strain, the greater the internal resistance of the rubber molecules in the viscous flow, which is due to the destruction of the carbon black filler network under the condition of high strain, resulting in weakening of the filler interaction. The amplitude of the decrease in G‘ gradually becomes smaller than the amplitude of the decrease in G′, which means that the rubber molecules need to consume more energy to overcome this resistance during the flow. Under the same strain conditions, the order of tanδ for the natural rubber compounds is roughly as follows: H-4 > H-5 > H-2 > H-1 > H-3. This indicates that when the ratio of PR107, Reyan72059, and Reyan73397 fresh latex is 1:1:3, the internal resistance of rubber molecules in the viscous flow of the H-3 compound is the smallest, which may be due to the appropriate mixing ratios of different varieties of fresh latex, the proportion of large and small rubber particles, and the content of non-rubber components in natural rubber to promote the dispersion of carbon black in the rubber and reduce the internal resistance of rubber molecules. This resistance requires less energy consumption [3,31,32,33].

**Figure 6:**
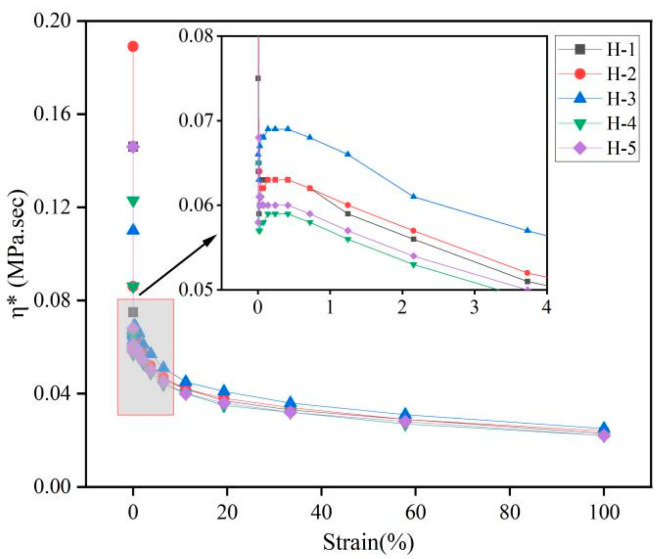
Responses of η* against strain of natural rubber compound.

**Figure 7:**
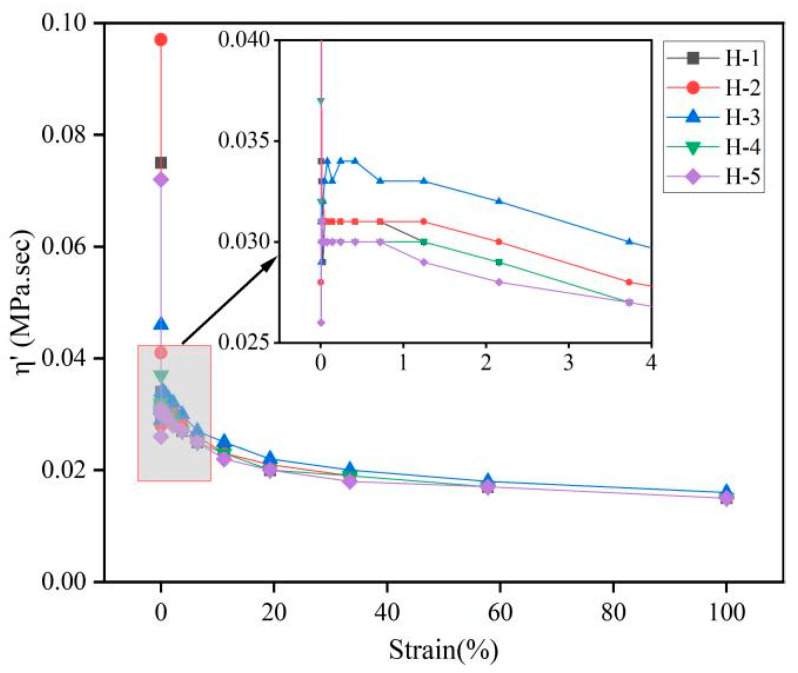
Responses of η′ against strain of natural rubber compound.

**Figure 8:**
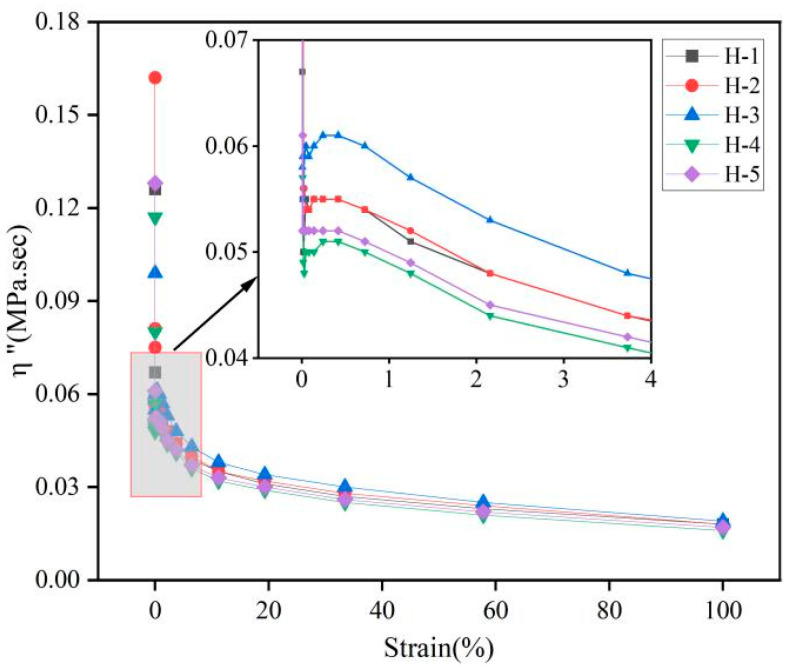
Responses of η″ against strain of natural rubber compound.

The changes in complex viscosity (η*), dynamic viscosity (η′), and imaginary viscosity (η″) of the natural rubber compound are shown below (Figure 6, Figure 7 and Figure 8). η* reflects the viscoelasticity [34,35] of the compound under oscillating shear stress or strain, and is calculated using the following Formula (1):

η* = η′ − iη″(1)

where η′ = G″/ω, which is related to the loss modulus and represents the viscous contribution, the energy dissipation part of the complex viscosity; i is the imaginary unit, which represents the imaginary part of the complex number; η″ = G′/ω, which is related to the storage modulus, and represents the contribution of elasticity and is the measure of elasticity and energy storage; and angular frequency (ω) represents the angular velocity of frequency. Its calculation Formula (2) is

Ω = 2πf(2)

where f represents frequency in Hertz (Hz); 2π is the coefficient for converting frequency from Hertz to angular frequency.

η′ reflects the ability of the compound to dissipate energy under oscillatory shear; η″ reflects the ability of the compound to store and release energy under oscillatory shear [36]. As can be seen from the figures, with the gradual increase in strain, the η*, η′, and η″ of the natural rubber compound gradually decreased and the change trend was the same, which is consistent with the change trend of its rubber G′, G″, indicating that the viscoelasticity of the natural rubber compound gradually decreased under high strain, which is because the carbon black filler network was destroyed under high strain, resulting in the weakening of the interaction between fillers. The energy dissipation increases, but the energy dissipation capacity decreases. Under the same strain conditions, the order of η*, η′, η″ of the natural rubber compound is roughly H-3 > H-2 > H-1 > H-5 > H-4, indicating that when the mixing ratio of PR107, Reyan72059, and Reyan73397 fresh latex is 1:1:3, the viscoelasticity of the carbon black compound is the highest.

### 3.5. Cross-Link Density of Natural Rubber Vulcanized Rubber

**Figure 9:**
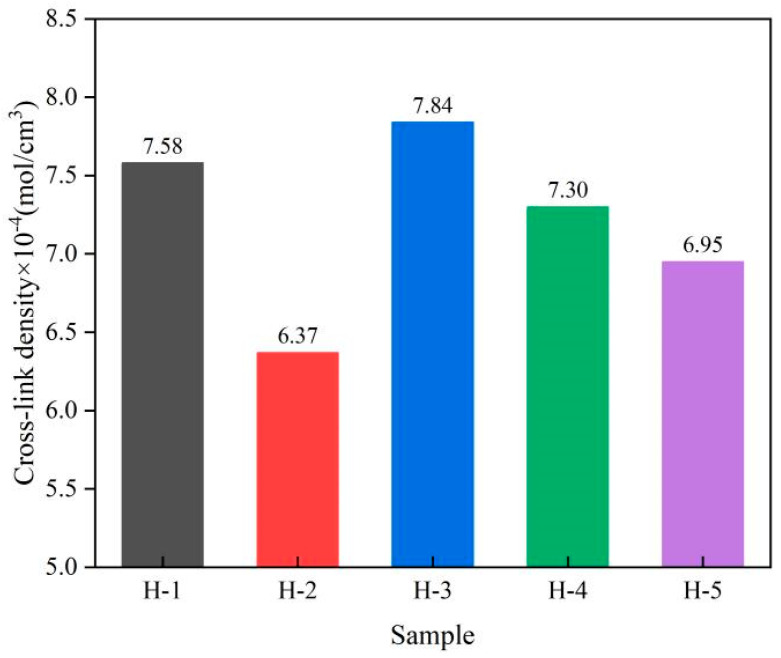
Cross-link density of natural rubber vulcanized rubber.

Cross-link density refers to the number of cross-linked bonds per unit volume of natural rubber, which is closely related to the properties of its rubber [27]. The change in cross-link density of natural rubber vulcanized rubber is shown below (Figure 9). As can be seen from the figure, the cross-link density of natural rubber vulcanized rubber does not show a clear trend, consistent with the trend of ∆M of the compound, further indicating that cross-link density is a measure of the degree of cross-link of natural rubber. Under the conditions of carbon black formulation, the cross-link density of H-1 vulcanized rubber is greater than that of H-2 vulcanized rubber, indicating that the degree of cross-link of acid-coagulated natural rubber vulcanized rubber is higher, which was attributed to the better dispersion of carbon black in acid-solidified natural rubber [37,38]. With the addition of PR107 and Reyan72059 fresh latex, the cross-link density of H-3 vulcanized rubber increased and reached the maximum value, which increased by 23.08% compared with H-2 vulcanized rubber, while the cross-link density of H-4 and H-5 vulcanized rubber also increased, but with the increase in the proportion of PR107 and Reyan72059 fresh latex, the cross-link density of vulcanized rubber decreased. This shows that under the same coagulation process conditions, the mixing of different varieties of fresh latex can improve the degree of cross-link of the vulcanized rubber. This may be due to the mixing of different varieties of fresh latex changing the proportion of rubber particles and the content of non-rubber components in natural rubber, which is more conducive to the cross-linking of rubber molecular chains [3].

### 3.6. Physical and Mechanical Properties of Natural Rubber Vulcanized Rubber

**Figure 10:**
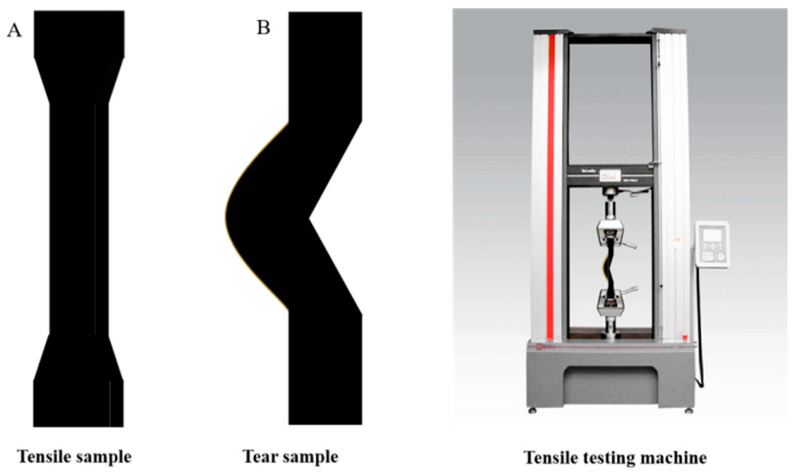
Testing of physical and mechanical properties of natural rubber vulcanized rubber. Tensile sample (**A**), tear sample (**B**) and testing of physical and mechanical properties of natural rubber vulcanized rubber.

The physical and mechanical properties of natural rubber vulcanized rubber were tested and changed as follows (Figure 10, Table 5). As can be seen from the table, under the conditions of carbon black formulation, the tensile strength and elongation at break of H-1 vulcanized rubber were higher than those of H-2 vulcanized rubber, consistent with the trend of cross-link density of the vulcanized rubber, indicating that acid-coagulated natural rubber has better compatibility with carbon black. Under the same coagulation process, the tensile strength, elongation at break, and tear strength of H-3 vulcanized rubber were the largest, and increased by 5.32%, 12.45%, and 3.70%, respectively, compared to H-2 vulcanized rubber, indicating that the proper mixing of different varieties of fresh latex was conducive to the improvement of the physical and mechanical properties of the vulcanized rubber. This is because the mixing of fresh latex changes the proportion of large and small rubber particles and the content of non-rubber components in natural rubber, increasing the dispersion of carbon black in rubber and improving the degree of cross-link of the vulcanized rubber [38,39]. With the increase in the proportion of PR107 and Reyan72059 fresh latex, the tensile strength and elongation at break of the vulcanized rubber gradually decrease, indicating that the mixing ratio of different varieties of fresh latex has a significant effect on the properties of the vulcanized rubber.

### 3.7. Heat Generation Performance of Natural Rubber Vulcanized Rubber

**Figure 11:**
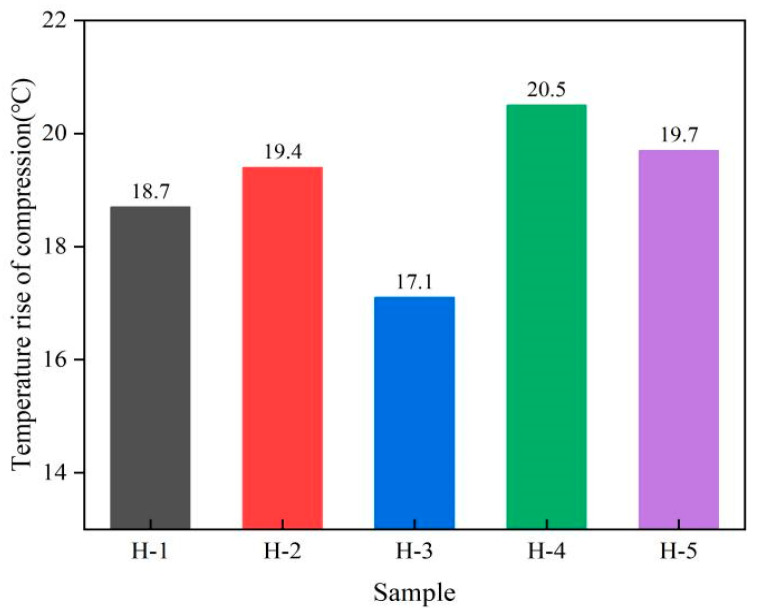
Heat generation performance of natural rubber vulcanized rubber.

Heat generation performance is an important index in natural rubber research. For the material under dynamic load after cyclic compression deformation, most of the consumed power is converted into heat energy, and the formation of compression heat generation performance has an impact on the use of products, life, and so on. The change in heat generation performance of natural rubber vulcanized rubber is shown as follows (Figure 11). As can be seen from the figure, under the conditions of carbon black formulation, the heat generation performance of H-1 vulcanized rubber is lower than that of H-2 vulcanized rubber, indicating that acid-coagulated natural rubber vulcanized rubber generated heat to a relatively lower degree during compression. When the ratio of PR107, Reyan72059, and Reyan73397 fresh latex is 1:1:3, the heat generation performance of the vulcanized rubber is the smallest, being reduced by 11.86% compared with H-2 vulcanized rubber, indicating that the mixture of different varieties of fresh latex can effectively improve the heat generation performance of the vulcanized rubber. This may be due to the good dispersion of carbon black in the compound prepared by mixing different varieties of fresh latex: the cross-link density of vulcanized rubber is large, and the molecular chain movement is limited [3,38].

### 3.8. Wear Resistance of Natural Rubber Vulcanized Rubber

**Figure 12:**
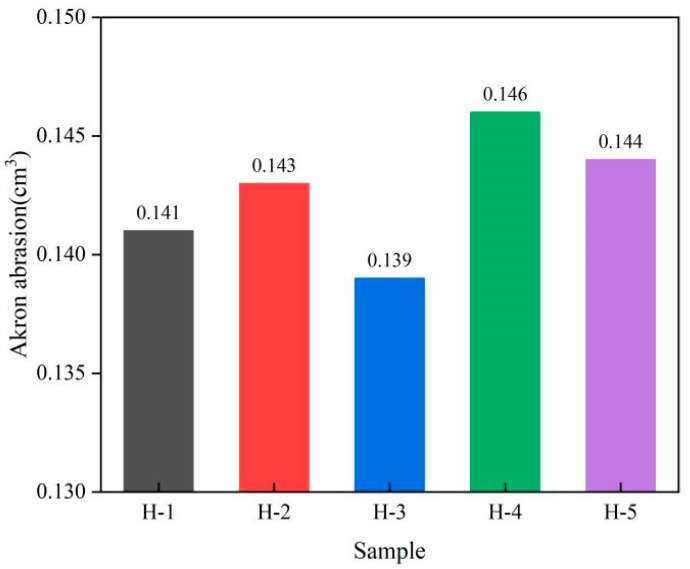
Akron abrasion of natural rubber vulcanized rubber.

Abrasion is a microscopic breakage and shedding phenomenon that occurs on the surface of rubber due to friction, and wear resistance is an important technical index for rubber products. Akron abrasion is a measure of the strength of rubber’s resistance to rolling friction. The change in wear resistance of natural rubber vulcanized rubber is shown below (Figure 12). As can be seen from the figure, the wear resistance of H-1 vulcanized rubber is slightly less than that of H-2 vulcanized rubber, indicating that the wear resistance of acid-coagulated natural rubber vulcanized rubber is better, which is due to the uniform dispersion of carbon black in acid-coagulated natural rubber and has a relatively good reinforcing effect on its vulcanized rubber. With the addition of PR107 and Reyan72059 fresh latex, the wear resistance of the vulcanized rubber decreases, indicating that the mixing of different varieties of fresh latex can improve the wear resistance of vulcanized rubber, which may be because the mixing of different varieties of fresh latex is conducive to the dispersion of carbon black in the rubber [38,40]. However, with the increase in the proportion of PR107 and Reyan72059 fresh latex, the wear resistance of the vulcanized rubber increases, indicating that improving the wear resistance of the vulcanized rubber requires controlling the mixing ratio of different varieties of fresh latex in a suitable range.

## 4. Conclusions

In this study, using acid-coagulated and enzyme-assisted microbial coagulated single-variety natural rubber as controls, the effects of the mixing ratio of PR107, Reyan72059, and Reyan73397 fresh latex on the processing and dynamic properties of bio-coagulated natural rubber were systematically analyzed. The results showed that the aging resistance of enzyme-assisted microbial coagulated natural rubber decreased, but increased with the increase in the mixing ratio of PR107 and Reyan72059 fresh latex. Compared with acid-coagulated natural rubber, the proportion of high molecular weight of natural rubber coagulated by the enzyme-assisted microbial increased, and the mixing of fresh latex had little effect on the molecular weight distribution curve. Under the carbon black formulation, the enzyme-assisted microbial coagulated natural rubber compound exhibited a higher CRI than the acid-coagulated natural rubber compound. Under the same strain conditions, the H-3 compound (PR107:Reyan72059:Reyan73397 = 1:1:3) had the best viscoelasticity and the least internal resistance of rubber molecules. Compared with the control sample H-2 vulcanized rubber, the cross-link density, tensile strength, elongation at break, and tear strength of H-3 vulcanized rubber significantly increased and reached the maximum, while the heat generation performance significantly decreased and became minimal, and the wear resistance improved, indicating that adjusting the mixing ratio of different varieties of fresh latex can significantly improve the processing properties, physical and mechanical properties, and wear resistance of natural rubber, and the heat generation performance can be reduced. This study investigated the intrinsic relationship between the ratio of fresh latex and the performance of natural rubber, which is of great significance for optimizing the processing of natural rubber and improving the comprehensive performance of the product. At the same time, the study can analyze the mechanism of the influence of fresh latex ratio on the performance of natural rubber from the molecular level, revealing the intrinsic nature of changes in the performance of natural rubber, which will provide a solid theoretical basis and strong technical support for the precise regulation of natural rubber performance. This not only helps to develop natural rubber with excellent performance, but also opens up new ideas for the research and development of new processing technologies for high-end products, accelerates the localization process of high-end products, reduces the dependence on imported natural rubber, and injects new vitality into the high-quality development of China’s natural rubber industry.

## Figures and Tables

**Table 1:** Ratio and coagulation method of fresh latex.

Sample	Variety	Ratio	Coagulation Method	Enzyme Amount (%, *w*/*w*)	Microbial Amount (%, *w*/*w*)
H-1	73397	0	Acid coagulation	0	0
H-2	73397	0	Enzyme-assisted microbial coagulation	0.05	10
H-3	107:72059:73397	1:1:3	Enzyme-assisted microbial coagulation	0.05	10
H-4	107:72059:73397	1:1:2	Enzyme-assisted microbial coagulation	0.05	10
H-5	107:72059:73397	1:1:1	Enzyme-assisted microbial coagulation	0.05	10

Note: fresh latex ratio, in terms of latex mass; enzyme dosage, in terms of dry gel content; microbial coagulant dosage, in terms of fresh latex mass.

**Table 2:** The carbon black formulations of natural rubber.

Formulation	Raw Rubber	Zinc Oxide	Sulfur	Stearic Acid	Carbon Black	TBBS ^1^
Mass, g	100	5.0	2.25	2.0	35.00	0.70

Preparation of natural rubber vulcanized rubber. ^1^ N-tert-butyl-2-benzothiazolinesulfonamide, powder, ether or ethanol insoluble content should be less than 0.3% (mass fraction). It should be stored in an airtight container at room temperature and tested for ether or ethanol insolubles every six months. If this content exceeds 0.75% by mass, the material should be discarded or recrystallised.

**Table 3:** Physical and chemical indexes of natural rubber.

Sample	P_0_	PRI %	M_L(1+4)_
H-1	39.73 ± 0.61	83.7 ± 0.75	81.67 ± 0.58
H-2	45.43 ± 0.65	77.2 ± 0.82	95.00 ± 1.00
H-3	44.97 ± 0.31	77.37 ± 0.4	92.00 ± 0.00
H-4	46.40 ± 0.53	78.5 ± 0.89	93.67 ± 0.58
H-5	46.30 ± 0.36	82.1 ± 0.46	92.67 ± 1.53

**Table 4:** Vulcanization characteristics of the natural rubber compound.

Sample	M_L_/dN·m	M_H_/dN·m	∆M/dN·m	t_10_/min	t_90_/min	CRI/s^−1^
H-1	2.07	14.79	12.72	2.73	9.30	0.25
H-2	2.43	14.72	12.29	2.90	9.35	0.26
H-3	2.16	14.96	12.80	2.67	8.77	0.27
H-4	2.08	14.73	12.65	2.85	9.27	0.26
H-5	2.45	14.95	12.50	2.92	9.35	0.26

Note: ∆M = M_H_ − M_L_; CRI = 100/(t_90_ – t_10_).

**Table 5:** Physical and mechanical properties of natural rubber vulcanized rubber.

Sample	Tensile Strength /MPa	100% Modulus /MPa	300% Modulus /MPa	500% Modulus /MPa	Elongation at Break /%	Permanent Set/%	Tear Strength /(kN/m)
H-1	27.63 ± 0.44	1.87 ± 0.02	7.70 ± 0.12	17.46 ± 1.09	667.21 ± 11.92	26.67 ± 0.74	88.49 ± 1.81
H-2	26.52 ± 0.84	2.55 ± 0.11	7.64 ± 0.14	20.6 ± 0.72	628.41 ± 14.68	26.44 ± 0.45	90.05 ± 1.17
H-3	27.93 ± 0.41	1.82 ± 0.10	7.48 ± 0.12	17.17 ± 0.32	706.67 ± 4.58	28.81 ± 0.99	93.51 ± 0.85
H-4	27.44 ± 0.41	1.83 ± 0.08	7.44 ± 0.06	17.16 ± 0.52	660.06 ± 16.67	30.31 ± 0.88	78.43 ± 0.81
H-5	26.92 ± 0.56	2.45 ± 0.13	10.72 ± 0.85	23.63 ± 0.45	536.69 ± 14.79	28.15 ± 0.8	80.13 ± 1.41

## Data Availability

The original contributions presented in this study are included in the article. Further inquiries can be directed to the corresponding author.
